# Impact of environmental sustainability on open innovation in SMEs: An empirical study considering the moderating effect of gender

**DOI:** 10.1016/j.heliyon.2023.e20096

**Published:** 2023-09-13

**Authors:** Paul Sarango-Lalangui, Mauricio Castillo-Vergara, Omar Carrasco-Carvajal, Antonio Durendez

**Affiliations:** a Department of Business Science, Universidad Técnica Particular de Loja (UTPL), Loja, Ecuador; b Faculty of Economy and Business, Universidad Alberto Hurtado, Santiago, Chile; c School of Engineering of the Faculty of Engineering and Architecture of the Universidad Central de Chile, Santiago, Chile; d Department of Economics, Accounting and Finance, Universidad Politécnica de Cartagena, Calle Real, 3, 30201, Cartagena, Spain

**Keywords:** Environmental sustainability, Innovation, Strategy, Open innovation, SMEs, Gender gap

## Abstract

Sustainable development has emerged as a crucial factor enhancing the competitiveness of small and medium enterprises (SMEs) in light of societal expectations and government regulations. Within this context, innovation assumes a significant role in this challenge, particularly for smaller companies facing greater obstacles in fostering innovation. These constraints have prompted both internal and external innovation initiatives, commonly referred to as open innovation. In this article, we scrutinize the influence of environmental practices on innovation endeavors, specifically examining whether the innovation strategy and its implementation facilitate the advancement of open innovation within SMEs. Furthermore, we explore the potential moderating effect of firm owners' gender on these relationships. The research model is validated using data from 543 companies in Ecuador, employing the partial least squares (PLS) method. The findings elucidate how environmental sustainability can promote the adoption of open innovation practices, with the innovation strategy and its implementation acting as mediators in this relationship. The impact of gender only manifests in the innovation strategy pertaining to environmental sustainability. In conclusion, open innovation stemming from the pivotal role of environmental sustainability emerges as a critical driver in augmenting innovation performance across various dimensions, such as quality, growth, efficiency, process enhancement, organizational practices, employee motivation, and acquisition of new product/service skills.

## Highlights

• Sustainable development enhances SME competitiveness.
• Innovation is crucial for overcoming SME innovation challenges.
• Environmental practices drive open innovation adoption.
• Gender influences innovation strategy based on sustainability.
• Open innovation drives performance across multiple dimensions.

## 1 Introduction

Owing to societal expectations and government regulations, sustainable development has emerged as a pivotal determinant of SME competitiveness [1]. For SMEs, the imperative to adopt socially responsible environmental behavior to reap benefits remains unquestionable. Consequently, sustainability management has become a formidable challenge for SMEs. Engaging in practices associated with sustainable development yields a plethora of advantages for companies, bolstering their competitive edge [2]. These benefits encompass waste reduction, cost savings, heightened customer satisfaction, increased employee involvement, superior product quality, and improved public relations [3]. Notably, an organization's sustainability strategy piques the interest of managers, professionals, and academics, contributing to the equilibrium of sustainability, as posited by the natural-resource-based view (NRBV). The NRBV extends the purview of the resource-based view and posits that companies adhering to environmentally sustainable strategies can attain a sustained competitive advantage. In particular, these firms amalgamate three interdependent practices: pollution prevention, product stewardship, and sustainable development [4].

The emergence of new socially responsible purchasing behaviors has compelled companies to adopt novel environmentally sustainable business models [5]. In this context, an open innovation model is pivotal in capitalizing on experiences related to environmental practices within organizations. Companies seeking to produce products with minimal environmental impact can leverage open innovation [6]. The open innovation model accentuates that companies can foster more effective innovations through external collaboration and internal knowledge networks [7]. Moreover, when environmental practices align with companies' innovation strategies, they can enhance business performance [8]. Establishing a correlation between economic and environmental performance is imperative [9].

On the other hand, there are relevant considerations for sustainability engagement in SMEs that are not “small big business” and behave differently than large corporations [10]. The impact of SMEs on environmental well-being may be even greater than that of large firms, given the volume of firms of this size [11]. Most sustainability studies focus on large companies and multinational organizations, neglecting the environmental and social impacts of small and medium-sized enterprises [12]. Furthermore, studies have focused on economic and management issues, ignoring sustainable practices and their impact on financial performance [13].

Existing literature has identified a research gap, necessitating a comprehensive exploration of the relationship between innovations and the adoption of environmental practices [14,15]. Notably, the focus should be on sustainability-oriented innovations as a business driver to improve performance and confer a competitive advantage [16]. Furthermore, future research should also incorporate cultural contexts and relational variables [17]. Scholars in this field acknowledge the significance of the NRBV in assessing firms' innovation activities, recognizing the connection between innovation and the differentiation of competitive advantages achieved through green capabilities. The traditional RBV fails to account for the competitive advantages firms can derive from a sustainable business strategy [18].

To address this gap, in this study, we examine the influence of environmental practices on the open innovation activities of SMEs and ascertain whether the innovation strategy and its implementation mediate the promotion of open innovation. The key research questions are as follows: Do environmental practices influence the open innovation activities of SMEs? Is this influence mediated by the innovation strategy and its implementation? Moreover, considering the particular interest in understanding the origins of the “gender gap” inequality in corporate governance [19], we pose the following questions: Does the gender of owners moderate the relationship between environmental sustainability, open innovation, and innovation performance? Based on a sample of 543 Ecuadorian SMEs, in this study, we analyze the interrelationships using a structural equation system. The examination of SMEs in Ecuador is of particular interest, as open innovation serves as a valuable strategy to enhance economic performance, environmental impact, and social responsibility. By involving multiple stakeholders the development of innovative solutions, SMEs leverage diverse ideas, resources, and knowledge to enhance their efficiency, sustainability, and responsiveness to market demands. Furthermore, open innovation assists SMEs in complying with increasingly stringent environmental and social standards and regulations while bolstering their reputation and stakeholder relationships. In Ecuador, productive sectors continually strive to contribute substantially to GDP, improving the business landscape and enabling companies to globalize in the market [20]. However, increased industrial production often comes at the expense of diminished social value and adverse environmental consequences. Such a tradeoff is being challenged in Ecuador by the emergence of ecologically minded entrepreneurs [21].

The remainder of this article is structured as follows. First, a comprehensive review of the literature on the influence of environmental practices and open innovation activities is conducted, and research hypotheses are proposed. Subsequently, the methodology, sample characteristics, and variables used are presented. Then, the obtained results are analyzed. Finally, we conclude the research by presenting the findings derived from this study.

## 2 Literature review and hypothesis

Sustainable development has garnered recognition as a competitive strategy adopted by companies [22] and is defined as an "organization's capacity to sustain its activities indefinitely, while considering its impact on capital, nature, society, and humanity” [23]. Sustainable development encompasses three pillars: economic, social, and environmental development. Environmental sustainability represents the third dimension within the realm of sustainable development [24]. The significance of sustainable development in the realm of business continues to intensify, permeating nearly all aspects of business operations [25]. Drawing on the perspective of the NRBV, companies can cultivate valuable and resource-intensive environmental capabilities that confer a sustainable competitive advantage [4]. SMEs account for approximately 90% of the world's enterprises and generate employment for 50%–60% of the world's population, playing an important role in environmental conservation [26]. SMEs are prominent in the sustainable development agenda because they contribute to economic growth and poverty reduction [27]. Sustainable development practices are recognized as a competitive business strategy [28,29] and generate expectations in society and government regulation [30]. Sustainability (the appropriate combination of economic, environmental, and social aspects) of SMEs prioritizes economic performance, considering environmental and social aspects to remain competitive [31]. Determining the appropriate management system to ensure sustainable development is therefore an important issue for SMEs [32], and a practical framework is required to identify and implement sustainable development plans [29], as well as to recognize the specific factors blocking their practice in small businesses [10].

### 2.1 Environmental sustainability and open innovation

Over the past five decades, environmental challenges have proliferated alarmingly, including record carbon emissions, energy waste, widespread pollution, inadequate wastewater management, and water scarcity. These problems have led to an accelerated increase in natural resource consumption, culminating in 2020 as the warmest year on record [[33], [34], [35], [36]]. Small and medium enterprises (SMEs), which are recognized as critical pillars in generating employment and driving global economic growth, are responsible for 60%–70% of global pollution [37,38]. However, the strategies implemented by SMEs to address these environmental challenges are still not adequately recognized and documented [39,40].

A company's ability to thrive sustainably over time is contingent upon its relationships with key stakeholders [23], which entail envisioning shared needs and fostering enhanced collaboration and satisfaction [22,41] and serving the interests of employees, current clients, and potential clients [42]. Sustainable development prompts a re-evaluation of innovation and technological advancements [43]. By embracing sustainability measures, companies gain competitive advantages through access to new markets and alignment with customer preferences [44]. Furthermore, responsible environmental behavior positively impacts innovation endeavors [45] and increases patent numbers [46]. Conversely, open innovation (OI) represents a deliberate process whereby companies seek and assimilate knowledge from external sources [[47], [48], [49]]. OI emerges through interactions with clients, suppliers, universities, public research institutions, and competitors [50,51]. Through the OI process, companies capitalize on their internal knowledge [49,52] while leveraging that knowledge externally [53], thereby fostering networks to seize opportunities [54].

Based on the reasoning presented above and the results of the research questionnaire, with our hypothesis, we posit that environmental practices developed by SMEs in terms of protection of the environment (conservation, climate change, pollution prevention, and biodiversity) and reduction in waste of raw materials, water, and energy have a positive impact on open innovation activities of SMEs. Therefore, following a pro-environmental business strategy simultaneously fosters open innovation activities, such as acquisition of knowledge from outside the company (customers, research institutions, external networks, and universities), the involvement of employees in R&D initiatives, exploitation of patents and royalties, and synergies and alliances with competitors. Given this background, the following hypothesis is proposed:

H1 Enhanced environmental practices enhance the open innovation activities of SMEs.

### 2.2 Innovation strategy, open innovation, and environmental sustainability

Innovation encompasses the creative design, enhancement, or invention of new products or services to achieve improved functional and economic outcomes. The ultimate objective of innovation is to advance knowledge and enhance sustainable growth [55]. The implementation of innovation strategies is contingent upon how such strategies are executed [56]. Hence, companies that adopt open innovation (OI) operate within external contexts that frequently influence their innovation and sustainability opportunities [57]. Environmental sustainability acts as a moderator, influencing the effects of OI on innovation performance. Likewise, companies can maintain competitiveness when environmental uncertainty is low without fundamentally redefining existing ecological knowledge [58].

Consequently, companies are inclined to introduce innovative and environmentally conscious products into the market, leveraging existing routines and knowledge [59]. Such an inclination leads companies to seek out existing eco-friendly products rather than developing entirely new ones [60]. Conversely, research demonstrates that OI strategies positively correlate with innovation performance [14,[61], [62], [63]], primarily because such strategies enable companies to transcend their boundaries and enrich their knowledge base. Environmental uncertainty represents a contextual factor that significantly impacts the effectiveness of OI strategies [64].

Corporate social responsibility (CSR) facilitates companies in establishing deep, lasting, and trust-based relationships with diverse stakeholders. This quality can be instrumental in promoting corporate benefits, especially in the open and collaborative exploration of knowledge [[65], [66], [67]]. More precisely, CSR can be classified into two distinct categories: corporate CSR and philanthropic CSR [68]. Corporate CSR focuses on stakeholders linked by direct market exchanges and is related to the ethical obligations inherent in companies' core business operations. In contrast, philanthropic CSR emphasizes charitable activities, and its scope extends to stakeholders who do not have direct exchanges with companies.

Companies that engage in OI strategies under conditions of high environmental uncertainty have a relatively strong impetus to effectively leverage external resources with respect to the environment [69] and expand their environmental knowledge domain [70]. Consequently, companies are better equipped to foster the development of eco-innovation, meeting these requirements. Sustainability is paramount for SMEs, as it presents rewards and challenges. Furthermore, a heightened understanding of the use of non-renewable resources, such as fossil fuels, has underscored the need for communities to transition toward more sustainable products and processes. The objectives outlined in the sustainable development agenda entail an expanding array of governance policies embraced by public- and private-sector actors [71]. Since the publication of the Brundtland report in the 1980s, it innovation has been widely recognized to play a crucial role in achieving sustainable development goals. Subsequently, policymakers and academics have engaged in extensive discussions regarding governance mechanisms promoting innovation [[72], [73], [74]].

The arguments presented above led us to consider a research hypothesis based on the key role of innovation strategies developed by SMEs when assessing the connection between environmental practices and open innovation. In that sense, when SMEs develop a business strategy in which the mission and/or vision include an innovation goal, internal cooperation, customer satisfaction, quality of products, and the participation of skilled employees in innovation, this key innovative characteristic can mediate the link between environmental practices and the open innovation activities of SMEs. According to this logic, we propose the following hypothesis:

H2 The innovation strategy serves as a mediator between environmental practices and open innovation activities of SMEs.

### 2.3 Innovation implementation, open innovation, and environmental sustainability

Corporate social responsibility (CSR) explicitly targets primary stakeholders, embodying the ethical obligations that emerge when a firm enters interactions and transactions with such key entities [75]. Within the framework of instrumental stakeholder theory, corporate CSR not only facilitates the building of strong and constructive relationships with primary stakeholders but also enables the acquisition of critical resources for innovation directly from these entities. Corporate CSR contributes to increasing the active participation of key stakeholders in open innovation (OI), thereby strengthening collaboration and synergy in innovative processes [76,77].

The business ecosystem is dynamically driven by evolving consumer needs, rapid market development, and technological advancements. To remain innovative, firms actively seek practical approaches to adapt to these changes [51,78]. One primary approach is the adoption of open innovation (OI) practices that emphasize leveraging internal and external knowledge to foster innovation [79]. Similarly, integrating sustainability into supply chains necessitates a creation-focused approach [80]. Companies aiming to achieve sustainability within their supply chains must consider innovation as a means to address adverse environmental impacts. Thus, innovation and environmental sustainability involve the implementation of new or modified products, processes, and techniques to minimize negative environmental effects [81]. For sustainable innovation to have a meaningful impact, it must be distributed in the market or implemented in a manner that yields sustainable outcomes and reduces socioenvironmental harm. Multiple factors must be considered when pursuing sustainable innovation within an organization, such as recycling, waste management, and ecological design [82].

Innovation plays a pivotal role in achieving long-term sustainable development. Companies, supply chains, and nations can realize environmental sustainability objectives by embracing factors of open innovation [83]. Environmental sustainability innovation encompasses product and process innovations that employ technologies aimed at conserving energy, preventing pollution, recycling waste, and managing the environment [84]. Knowledge management and learning are vital in fostering innovation [85], as they drive technical progress and provide incentives for environmental sustainability in business contexts. In conclusion, innovation in environmental sustainability is recognized as a key factor in enhancing companies' environmental, social, and financial performance. Numerous initiatives in environmental sustainability are geared towards improving technological processes, gaining a competitive advantage, fostering open innovation, and reducing manufacturing costs [86].

In accordance with the arguments presented above, we consider a research hypothesis related to the key function of innovation implementation in the relationship between environmental practices and open innovation in the context of SMEs. When SMEs have a clearly defined strategy, innovation is planned formally with a long-term orientation, innovation activities are properly coordinated in conjunction with employees, and the implementation of innovation becomes a mediator between environmental standards and open innovation. Thus, the following hypothesis is proposed:

H3 The implementation of innovation acts as a mediator between environmental practices and open innovation activities of SMEs.

### 2.4 Open innovation and innovation performance

The existing literature demonstrates a positive impact of open innovation (OI) on various measures of company performance [[87], [88], [89]] and highlights the benefits that specifically affect the performance of small and medium-sized enterprises (SMEs) [90]. OI enables SMEs to tap into external sources of knowledge and ideas, reducing investment costs and sharing risks [91]. Incorporating external knowledge fosters innovation activities, accelerates the implementation of the innovation process, and enhances innovative performance [92]. OI is widely recognized as a crucial practice for achieving innovation performance [93]. OI activities improve a company's technological position, facilitate access to new markets [94], and expedite the introduction of innovations in the market [95]. Therefore, OI contributes enhances innovation performance and overall factor productivity [96]. Lastly, OI increases the likelihood of significant business growth and economic efficiency by generating revolutionary innovations [91]. According to above reasoning, promoting open innovation activities, such as acquisition of knowledge from outside the company (external sources), involvement of employees in R&D initiatives, exploitation of patents and royalties, and synergies and alliances with competitors, has a positive effect on the innovative performance of SMEs. The innovative performance of SMEs is a wide research construct that comprises the ability to introduce high-quality new products, increasing turnover, efficiency in delivery processes, improved organizational practices, creativity, skilled employees, and improved teamwork. Thus, the following hypothesis is proposed:

H4 Open innovation practices have a positive impact on the innovative performance of SMEs.

### 2.5 The moderating role of gender

The increasing participation of women in social and economic activities has prompted scholars to investigate how the gender of managers and owners influences organizational performance. Researchers, practitioners, and policymakers have recognized the importance of promoting economic activity and growth among women entrepreneurs [97]. According to post-structural feminist theory, gender inequality is diminishing as more managers and companies implement measures to promote women to senior management positions [98,99].

Despite progress, women are still less likely to succeed as entrepreneurs compared to men for various reasons, such as lack of support networks, financing obstacles, and limited participation in the labor force [99]. Although some gender-related limitations have been identified and studied, the relative influence of women in certain domains remains unresolved [100].

Fostering the proliferation of women-owned businesses may be a suitable approach to address institutional constraints and achieve organizational efficiency [101,102]. Both liberal and social feminist theories suggest that there may be differences in the ways in which women and men manage their businesses, such as women being more risk-averse [102]. Women in leadership positions, particularly as owner–managers, can overcome organizational challenges by demonstrating participative leadership and collaborating with managers rather than controlling them. They are more effective in addressing strategic issues, which can motivate managers to engage in long-term innovation projects [103]. Women's participation in the decision-making process can contribute to the achievement of strategic goals [104].

Women's role in innovation management is a complex task, but innovations are crucial drivers of change. Women can navigate these challenges through stakeholder relationships, leading to sustainable innovation [105]. Existing research suggests a strong correlation between women's management and innovation effectiveness [106]. Women owners can enhance innovation and the quality of decision-making processes.

Furthermore, based on their unique characteristics, female owners can provide executives with complementary knowledge and market information beyond their own experiences, resulting in more efficient innovation decisions [107]. Rather than solely focusing on growth, women emphasize business sustainability and stability by fostering harmonious relationships within the network of employees, suppliers, customers, and other stakeholders [101]. Previous studies have indicated that women express greater environmental concerns compared to men, suggesting a higher level of awareness of sustainability issues [108]. Women entrepreneurs are more likely to engage in social and environmental matters compared to their male counterparts, who tend to be more traditional and economically oriented [109].

According to the arguments presented above, the literature has considered the importance of including gender as a moderating variable in environmental research approaches [110,111]. Previous studies confirmed the significative moderating effect of gender when evaluating corporate environmental responsibility [111]. In particular, in the context of SMEs, gender diversity plays a relevant moderating role when analyzing the development of green business practices [112], as well as the connection between sustainable entrepreneurship and SME performance [113]. Based on the aforementioned reasoning, we expect that the presence of female owners moderates the relationship between environmental sustainability, open innovation, and innovation performance. Therefore, we propose the following hypothesis:

H5 The gender of owners has a moderating effect on the relationship between environmental sustainability, open innovation, and innovation performance of SMEs.

## 3 Methodology

### 3.1 Sample and data collection

In Ecuador, to initiate the deconcentrating and decentralization processes, the government published, by decree, Official Gazette No. 205 of June 2, 2010, declaring that the country would have nine planning zones composed of 140 districts and 1134 circuits. A proportion of 99% of the business sector comprises small and medium-sized companies, which due to their turnover, capital stock, number of workers, production level, or assets, have characteristics of this type of economic entity. According to the latest update of the statistical information system of the Directory of Companies and Establishments, there are 899,208 companies in Ecuador. The total number of companies includes all economic units that registered sales with the Internal Revenue Service (SRI). In the Ecuadorian economy, SMEs represent 90% of productive units, generate 60% of employment, and participate in approximately 50% of production. In addition, because they do not have many workers, SMEs are characterized by organizational structures that efficiently adapt to economic shocks. The most significant number of technological innovations and placements of national production in foreign markets are also attributed to SMEs.

The sample selection was based on the size classification of companies according to the number of employees, following the recommendation of the Oslo manual: companies with less than 10 employees, between 10 and 49 employees, and between 50 and 249 employees [114].

Data for the study were collected using a structured questionnaire designed to measure the latent variables of the proposed model and profile the respondents. Before completing the questionnaire, respondents read and accepted the provided informed consent. The measurement scales for each latent variable achieved internal validity by including items previously used in other research [115]. The questionnaire made reference to the indicators of the Ethos Institute, a Brazilian non-governmental organization focused on promoting sustainability in business strategies. The Ethos Institute's indicators represent a management tool for companies of all sizes and sectors to implement socially responsible management practices.

Before distributing the final questionnaires, presampling was conducted, which involved main executive directors who make company decisions and have expertise in innovation and sustainability. A total of 782 questionnaires were sent to CEOs, and 543 valid questionnaires were received, resulting in a response rate of approximately 69%. The sampling error was calculated to be ±3.50% at a confidence level of 95% (Z = 1.96, p = q = 0.5).

A 69% response rate was achieved in this study through a combination of strategies. First, companies were contacted in advance to highlight the importance of the study, increasing their willingness to participate. In addition, incentives such as access to the results and personalized followup, such as phone calls and reminder emails, were offered to ensure a high response rate.

Selection was performed through stratified random sampling based on criteria such as size and sector and through a contact list of chambers of commerce, business associations, and regulatory entities in Ecuador. The questionnaires were distributed by email and through telephone calls, depending on the accessibility and preferences of the target population.

Respondents completed the questionnaires online via Google Drive or email or by phone. The choice of method depended on the need to ensure data quality. In addition, it is common in research to follow up with participants who have yet to respond through email reminders, phone calls, or face-to-face visits to increase the response rate.

Furthermore, as a guideline, a minimum of 100 cases is recommended to achieve reasonable levels of statistical significance [116]. The sample size of 543 valid questionnaires in this study conforms with this guideline. Table 1 presents the sample characteristics, considering the economic activity of the included companies.

**Table 1:** Sample distribution. Sample size = 543.

Sector	Frequency	Average years in operation	Average % Female manager	Average % Female control in ownership	% Family-owned companies
Manufacturing	98 (18%)	17,96 years	21%	29%	56%
Construction	49 (9%)	16,65 years	18%	34%	57%
Research & Development	40 (7,4%)	19,12 years	15%	29%	32%
Commerce	240 (44,2%)	17,32 years	23%	36%	58%
Tourism	69 (12,7%)	18,33 years	19%	34%	59%
Services	45 (8,3%)	21,26 years	27%	33%	47%
Others	2 (0,4%)	15 years	100%	50%	100%

### 3.2 Variables

A quantitative research approach was employed for this study, and data were collected through a structured questionnaire. The questionnaire consisted of items adapted from existing scales or developed specifically for this research. All items were measured using a five-point Likert scale, ranging from 1 (totally disagree) to 5 (totally agree).

The following scales were used to measure the different dimensions:

1. Environmental sustainability: The scale proposed by Ethos was utilized to assess the association between technological innovation, environmental sustainability, and its impact on the performance of small businesses. Seven items were used to measure different aspects, such as conservation of the environment, climate, production, cleaning, pollution prevention, waste reduction, water use, energy consumption, and environmental maintenance.
2. Innovation strategy: The scale proposed in Ref. [117] was adapted to measure the innovation strategy dimension. Seven items were used to evaluate innovation vision, strategy, internal cooperation, customer satisfaction, product quality, employee skills, and employee engagement.
3. Innovation implementation: The scale proposed in Ref. [118] was adapted to measure innovation implementation. This dimension was assessed using five items related to innovation strategy, innovation activities, long-term innovation, and the application of creation, and execution of innovation.
4. Open innovation: The scale proposed in Ref. [119] was used to measure open innovation. Eight items were employed to evaluate internal knowledge, royalties, R&D, innovation process, external networks, company shares, purchase of services, and intellectual property.
5. Innovation performance: The scale proposed in Ref. [119] was used to measure innovation performance. Eleven items assessed dimensions new products, quality, sales increase, modified products, delivery processes, costs and time, organizational practices, motivation, qualification, teamwork, and promotion.

Regarding gender, company ownership was categorized based on the responses collected in the survey. Companies with female ownership exceeding 50% were classified as women-owned companies, following the approach suggested in Ref. [97].

In addition, three control variables were included to account for potential sources of variation. These variables were manager gender (coded as 1 for female and 2 for male), general manager age (measured in years), and company control (coded as 1 for family control and 2 for non-family control). Including these control variables helps to address potential endogeneity issues and is a commonly reported practice in the literature [120,121].

### 3.3 Analysis

The partial least squares structural equation modeling (PLS-SEM) technique was employed for data analysis. The Smart PLS version 4.0.8.3 statistical software developed by the authors of [122] was used for the analysis.

In recent years, structural equation modeling has become a powerful tool for multivariate analysis, and its use has become widespread in the social sciences [123,124], as an extension of traditional linear modeling and factor analysis techniques [125,126]. Its use is beneficial in social science research, where most concepts are not directly observable [127,128].

Two models were calculated: the measurement (outer) model, which establishes the relationships between the observed variables and latent variables, and the structural (inner) model, which determines the strength and direction of relationships between the latent variables [129]. Mode A, as recommended ins [130], was selected to handle the constructs.

Harman's single-factor test proposed in Ref. [116] was applied to address common method bias.

The data were generated according to a simplified version of the structural model [131], which consists of an exogenous variable ($\varepsilon_{1}$) and four endogenous variables ($\eta_{1}$, $n_{2}$, $n_{3}$, $\eta_{4}$). The values $\tau_{ij}$ and $\beta_{ij}$ correspond to the path effect in the model. The manifest variables are denoted x for the ε variables and y for the η variables. The software sets the $\pi_{i}{,x}_{i}$ values to optimize the process. The values $v_{1}$, y, and $\delta_{1}$ correspond to the error term.

Internal structure is defined as:

(1) $$\eta_{1}=\tau_{11}\varepsilon_{1}+v_{1}$$

(2) $$\eta_{2}=\tau_{12}\varepsilon_{1}+v_{2}$$

(3) $$\eta_{3}=\tau_{13}\varepsilon_{1}+\beta_{21}\cdot \eta_{1}+\beta_{31}\cdot \eta_{2}+v_{3}$$

(4) $$\eta_{4}=\beta_{41}\cdot \eta_{3}+v_{4}$$

Manifest variables are defined as:

(5) $$\varepsilon_{1}=\sum_{i=1}^{n}\pi_{i}x_{i}+\delta_{1}$$

(6) $$y_{i1}=\lambda_{i^{1}}\eta_{i}+\varepsilon_{i^{1}}$$

(7) $$y_{i2}=\lambda_{i^{2}}\eta_{i}+\varepsilon_{i^{2}}$$

(8) $$y_{i3}=\lambda_{i^{3}}\eta_{i}+\varepsilon_{i^{3}}$$

(9) $$y_{i4}=\lambda_{i^{4}}\eta_{i}+\varepsilon_{i^{4}}$$

The common method bias may potentially inflate the relationships between variables in the research because the same source collects information for both dependent and independent variables [132]. Bias is analyzed by applying Harman's single-factor test [133]; given problems concerning the common method, the variance factor analysis reveals that all variables are grouped into a single factor that explains much of the variance. The research results (KMO: 0.927; Bartlett's test of sphericity: Sig. 0.000) account for 75.101% of the total variance. The main factor explains 32.949% of the conflict, indicating that the common method variance bias is insignificant in the data.

To analyze possible endogeneity problems, we performed a Gaussian copulas analysis of the response variables [134,135].

A multigroup analysis was performed to test the moderating hypothesis (H5), following the approach described in Ref. [136]. Before comparing the path estimates between groups, it was essential to ensure the measurement invariance of the constructs. Such a step helps to confirm that the moderating effect of gender in the model is related to the structural model's trajectory coefficients rather than the external model's parameters. The three-step procedure for analyzing the measurement invariance of composite models (MICOM), as outlined in Refs. [137,138], was used in this study. The MICOM procedure was performed by running a two-tailed permutation test for the control variables (gender and sector) at a significance level of 5% for 10,000 permutations.

Overall, PLS-SEM, along with the tests and procedures described above, was employed to analyze the data and test the research hypotheses robustly and rigorously.

## 4 Results

### 4.1 Outer model

As Mode A was used as the estimation method in this study, we applied the traditional measures established to assess reliability and internal validity [139,140]. Table 2 shows the loads for each indicator and the values of Cronbach's Alpha, rho_A, composite reliability (CR), and the mean–variance extracted (AVE). The loads have values greater than 0.707, except for two indicators, but we decided to exclude them to improve the explanation of the construct [141]. Cronbach's alpha, rho_A, and CR are more significant than 0.70, confirming that all variables meet the reliability requirement of the construct. The AVE value exceeds the minimum value of 0.50 for each construct, showing the convergent validity of the measures. Table 3 shows the parameters that account for the achievement of discriminant validity [142].

**Table 2:** Values outer model.

Factor loadings	
Environmental sustainability. Cronbach's alpha: 0.923, rho_A: 0.940, Composite Reliability: 0.938, AVE: 0.682	
Does the company contribute to the conservation of the environment?	0.776
The company seeks to know the possible impacts of climate change on its business.	0.840
Is the company recognized for excellence in cleaner production and pollution prevention management?	0.829
Does the company take specific initiatives to reduce raw material waste?	0.837
Does the company look for efficient mechanisms for the use of water?	0.856
Does the company develop strategies, policies, or systems to optimize and reduce energy consumption?	0.853
We have excellent knowledge about maintaining nature (i.e., land, biodiversity, and ecosystem).	0.787
**Innovation Strategy.** Cronbach's alpha: 0.936, rho_A: 0.959, Composite Reliability: 0.947, AVE: 0.718	
The company's vision or mission includes a reference to innovation	0.822
The innovation strategy has helped achieve its strategic objectives	0.889
Internal cooperation is an essential part of strategy implementation	0.880
Customer satisfaction is part of our innovation strategy	0.886
The improvement of the quality of the product is one of our objective keys to the strategy	0.826
Formulating the innovation strategy increases the skills of employees	0.859
Improving employee engagement, morale, or both is part of following the innovation strategy	0.762
**Innovation Implementation.** Cronbach's alpha: 0.940, rho_A: 0.944, Composite Reliability: 0.954, AVE: 0.806	
We have a clearly defined innovation strategy	0.906
Innovation activities are planned formally and in writing	0.864
Long-term innovation activities are planned	0.912
The implementation of innovation activities is properly coordinated	0.907
Employees properly develop innovation activities	0.898
**Open Innovation.** Cronbach's alpha: 0.929, rho_A: 0.933, Composite Reliability: 0.942, AVE: 0.672	
Starting a new business from the internal knowledge of the company itself	0.767
Sale or offer of licenses or royalty agreements to other companies to obtain benefits from their intellectual property, patents, copyrights, or trademarks	0.846
Leverage the insights and initiatives of employees who are not involved in R&D (for example, by taking suggestions, exempting them from applying ideas, or creating autonomous teams to make innovations)	0.719
The direct participation of customers in their innovation process	0.753
Activities developed based on external networks to support innovation processes, being able to acquire external knowledge or human capital	0.894
Participation in new or established companies to gain access to their knowledge or to obtain other synergies	0.863
Purchase of R&D services from other organizations, such as universities, public research organizations, commercial engineers, or suppliers	0.850
The purchase or use of intellectual property, such as patents, copyrights, or trademarks of other organizations, to benefit from external knowledge	0.848
**Innovation Performance.** Cronbach's alpha: 0.921, rho_A: 0.948, Composite Reliability: 0.929, AVE: 0.544	
Ability to introduce new products and services to the market better than competitors	0.751
Quality of new products and services introduced	0.655
Increased sales generated by new products	0.811
Increased sales generated by the modified products	0.790
Efficiency in delivery processes inside and outside the work environment	0.657
Improved processes to save costs and time	0.726
Simplification of the operation betting on better organizational practices	0.739
Employee motivation to be more creative	0.768
Improvement in the qualification of employees	0.712
Improved teamwork	0.746
Greater possibility of promotion of employees thanks to innovation	0.744

**Table 3:** Discriminating validity.

Fornell-Larcker						Heterotrait-Monotrait Ratio (HTMT)				
	1	2	3	4	5	1	2	3	4	5
1. Environmental Sustainability	0.826	0.000	0.000	0.000	0.000	0.000	0.000	0.000	0.000	0.000
2. Innovation Strategy	0.199	0.847	0.000	0.000	0.000	0.188	0.000	0.000	0.000	0.000
3. Innovation Implementation	0.268	0.733	0.898	0.000	0.000	0.269	0.769	0.000	0.000	0.000
4. Open Innovation	0.302	0.516	0.595	0.820	0.000	0.314	0.517	0.630	0.000	0.000
5. Innovation Performance	0.295	0.340	0.248	0.329	0.738	0.303	0.334	0.222	0.293	0.000

### 4.2 Inner model

The structural model provides the results with respect to the hypotheses. A total of 5000 resamples (bootstrap techniques) were used to assess the statistical significance of the path coefficients [143]. Table 4 includes the trajectory coefficients that represent the direct effects of the exogenous constructs on the endogenous constructs, the confidence intervals of the coefficients, the t-value, and the value of f2. All proposed hypotheses were supported. Except for the control variables that were not supported.

**Table 4:** Values inner model.

Hypothesis	Path	Interval	p-value	t-value	f^2^	Hypothesis supported
Environmental Sustainability → Innovation Strategy	0.268	[0.222; 0.359]	0,000	6.435	0.077	Yes
Environmental Sustainability → Innovation Implementation	0.338	[0.285; 0.425]	0,000	7.996	0.129	Yes
Environmental Sustainability → Open Innovation	0.134	[0.065; 0.203]	0,001	3.172	0.030	Yes
Innovation Strategy → Open Innovation	0.372	[0.290; 0.460]	0,000	7.276	0.138	Yes
Innovation Implementation → Open Innovation	0.311	[0.223; 0.398]	0,000	5.790	0.092	Yes
Open Innovation → Innovation Performance	0.487	[0.445; 0.574]	0,000	12.515	0.311	Yes
Control Family Business → Open Innovation	0.045	[−0.092; 0.189]	0.533	0.623	0.001	No
Control Family Business → Innovation Performance	0.215	[−0.007; 0.443]	0.062	1.868	0.014	No
Control Gender Manager → Open Innovation	−0.054	[−0.221; 0.103]	0.511	0.657	0.001	No
Control Gender Manager → Innovation Performance	0.037	[−0.186; 0.258]	0.740	0.332	0.000	No

**Fig. 1:**
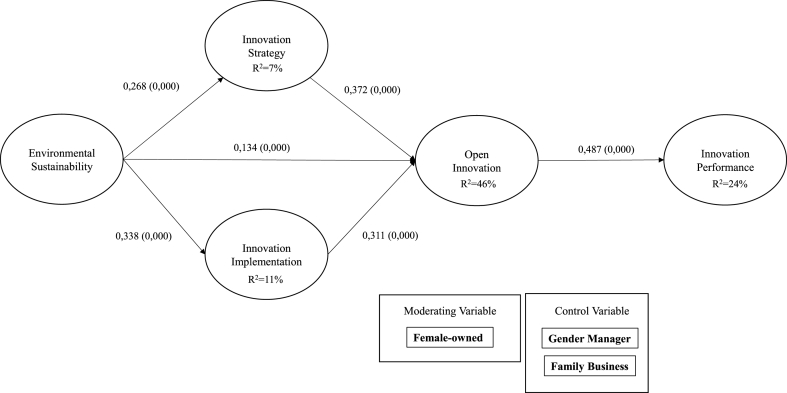
Structural model and results.

Based on the evaluation of the estimated model, the goodness of fit was assessed using the method proposed in Ref. [144]. The model's standardized root mean square residual (SRMR) was 0.074, which is below the recommended threshold of 0.10. The model explains 46.5% of the variance in open innovation, 23.7% of the variance in innovation performance, and 11.4% of the variance in innovation implementation, indicating weak but acceptable levels of explanation [125,145]. Fig. 1 illustrates the model and its results. Table 5 presents the data from the endogeneity analysis of Gaussian Copulas, reporting no endogeneity problems in the model. They were tested in all the proposed relationships.

**Table 5:** Gaussian copulas.

	Original Sample	Statistical t (|O/STDEV|)	p-value
GC (Open Innovation) → Innovation Performance	0.168	0.725	0.469
GC (Innovation Implementation) → Open Innovation	0.001	0.007	0.995
GC (Environmental Sustainability) → Innovation Strategy	0.116	0.681	0.496
GC (Environmental Sustainability) → Innovation Implementation	0.099	0.629	0.529
GC (Environmental Sustainability) → Open Innovation	−0.059	0.469	0.639
GC (Innovation Strategy) → Open Innovation	−0.356	1.423	0.155

## 5 Discussions

The results confirm the research hypotheses for small and medium-sized enterprises (SMEs) in the context of Ecuador, aligning with the emergence of environmentally conscious entrepreneurs [21] and the efforts of productive sectors to significantly contribute to GDP, enhance the business environment, and globalize companies in the market [20]. The significant association between environmental sustainability and innovation aligns with previous findings reported in literature in various cross-cultural contexts, including Austria [14], the Netherlands [16], and Brazil [70]. The results demonstrate a significant connection between environmental sustainability and innovation strategy (path: 0.268; p < 0.01), environmental sustainability and innovation implementation (path: 0.338, p < 0.01), and environmental sustainability and open innovation (path: 0.134, p < 0.01).

Furthermore, the results indicate mediating effects of innovation strategy (path: 0.372; p < 0.01) and innovation implementation (path: 0.311, p < 0.01) in the relationship between environmental sustainability and open innovation, suggesting that adopting environmentally sustainable practices, coupled with the development and implementation of a clear innovation strategy, can promote open innovation. According to the natural-resource-based view (NRBV) framework, the findings indicate that SMEs embracing green practices and capabilities within their organization can leverage both direct and indirect paths to achieve a competitive advantage in terms of innovation capacity. Open innovation is particularly crucial for SMEs, as it allows them to harness internal (know-how and R&D) and external (customers, suppliers, competitors, and R&D institutions) knowledge and resources to drive innovative outcomes. In the modern competitive landscape, managing innovation in isolation from stakeholders' influence is deemed impossible [146,147].

Lastly, fostering open innovation in SMEs is key to enhancing innovation performance. The results highlight that open innovation is a crucial driver of innovation performance in various aspects, such as the quality of new products/services, growth, efficiency, process improvements, organizational practices, employee motivation, and skills.

Our research shows a positive relationship between environmental sustainability and open innovation, the results of which are consistent with those reported in other studies [148]. Thus, the literature shows that management practices positively impact innovation and technology in SMEs [[149], [150], [151]]. Innovation strategy plays a relevant role in improving open innovation practices, the results of which are consistent with several studies conducted by other authors in other countries [[152], [153], [154]], facilitating the business model [155]. The results indicate that open innovation practices positively impact the innovation performance of SMEs, consistent with results reported by other authors [[156], [157], [158]]. Our results validate the worldwide literature, which indicates that SMEs reach new markets earlier in association with such practices [159]. Sustainable development has a positive relationship with innovation implementation, the result of which validate the findings of other studies [160,161].

The results of the multigroup analysis (MGA) based on permutations are shown in Table 6 and Table 7. Concerning gender participation in company ownership, only one of the relationships shows statistically significant differences: the effects of environmental sustainability and the implementation strategy are lower in companies with higher female participation (path difference: −0.166 p < 0.05). The results do not indicate an unobserved level of heterogeneous bias because the model results did not change when analyzing groups with control variables. Therefore, hypothesis 2 is partially supported.

**Table 6:** MICOM results.

Composite	Original Correlation	5.0%	p-value	Compositional invariance?
1. Environmental Sustainability	0.997	0.994	0.191	Yes
2. Innovation Strategy	1	0.996	0.910	Yes
3. Innovation Implementation	1	0.999	0.755	Yes
4. Open Innovation	0.999	0.999	0.246	Yes
5. Innovation Performance	0.985	0.973	0.153	Yes
Composite	Difference in mean value	95% confidence interval	p-value	Equal mean values?
1. Environmental Sustainability	0.054	[-0.179; 0.177]	0.549	Yes
2. Innovation Strategy	−0.155	[-0.177; 0.176]	0.085	Yes
3. Innovation Implementation	−0.203	[-0.176; 0.176]	0.024	No
4. Open Innovation	−0.165	[-0.178; 0.179]	0.071	Yes
5. Innovation Performance	−0.112	[-0.178; 0.174]	0.213	Yes
Composite	Difference in variances ratio	95% confidence interval	p-value	Equal variances?
1. Environmental Sustainability	0.041	[-0.224; 0.216]	0.715	Yes
2. Innovation Strategy	−0.071	[-0,295; 0.265]	0.615	Yes
3. Innovation Implementation	0.077	[-0.267; 0.253]	0.556	Yes
4. Open Innovation	−0.057	[-0.244; 0.231]	0.623	Yes
5. Innovation Performance	−0.158	[-0.288; 0.281]	0.279	Yes

**Table 7:** Permutation-based multigroup analysis for path coefficients and indirect effects.

Hypothesis	Path	Path Coefficients (female-owned = (1))	Path Coefficients (male-owned = (0))	Path Coefficients Difference	Permutation p-value	Hypothesis Supported
H5	Environmental Sustainability → Innovation Strategy	0.101	0.268	−0.166	0.045	Yes
	Environmental Sustainability → Innovation Implementation	0.223	0.307	−0.084	0.288	No
	Environmental Sustainability → Open Innovation	0.149	0.173	−0.024	0.787	No
	Innovation Strategy → Open Innovation	0.255	0.118	0.136	0.253	No
	Innovation Implementation → Open Innovation	0.449	0.416	0.033	0.797	No
	Open Innovation → Innovation Performance	0.238	0.384	−0.147	0.054	No

The results of the MICOM procedure for the sector variable indicate that the multigroup analysis does not make sense, given the absence of measurement invariance. In addition, we controlled the sector variable for the open innovation and innovation performance variables, showing no significant effect.

Our findings confirm some differential behavior according to the gender of the owner with respect to strategic innovation decision-making processes [103] and environmentally sustainable policies [109] in SMEs. The results show that women owners exhibit specific behavior that can reduce the effect of environmentally sustainable policies on the innovation strategy of SMEs. Previous research confirmed that female entrepreneurs have differential attitudes and managerial styles [162]. According to previous literature, they are more effective with respect to strategic issues that could motivate managers to engage in long-term innovation projects [103], and their participation in the decision-making process can contribute to the achievement of strategic goals [104]. However, other studies highlight differences in terms of risk taking, implying the need for caution with respect to some strategic management decisions, such as those concerning growth [147]. The lack of more significant results implies the need to further extend our initial results with respect to the role of gender in the context of environmental and OI decisions in SMEs.

## 6 Conclusions

In this study, we investigated how environmental sustainability can facilitate open innovation practices in small and medium-sized enterprises (SMEs), the mediating role of innovation strategy and implementation, and their impact on innovation performance. We surveyed 543 Ecuadorian SMEs from various industries. The findings reveal a significant positive correlation between environmental sustainability and open innovation approaches.

The results suggest that the innovation strategy can act as a bridge between environmental sustainability and open innovation in the case of SMEs. Additionally, this study highlights that both innovation strategy and implementation play intermediary roles in the relationship between environmental sustainability and open innovation practices.

According to the natural-resource-based view (NRBV) framework proposed in Ref. [4], companies that have developed a competitive advantage by implementing environmental sustainability practices are more likely to engage in innovation activities, generate new patents, and better serve the interests of customers and employees. Environmentally conscious SMEs are inclined to adopt green practices, such as waste reduction, water efficiency measures, energy optimization, cost reduction strategies, and proactive measures to address climate change impacts. Such behaviors enable SMEs to develop an environmentally sustainable business model that promotes open innovation.

The findings reported herein also indicate a mediating effect of innovation strategy and implementation in the relationship between environmental sustainability and open innovation in SMEs, suggesting that a clear innovation strategy and effective implementation can enhance the positive association between open innovation and sustainable green practices. Within the NRBV framework, the results of this study demonstrate that SMEs embracing green practices and capabilities within their organization can leverage both direct and indirect effects to gain a competitive advantage in terms of innovation capacity. Open innovation, which involves leveraging both internal and external knowledge and resources, positively impacts the innovation performance of SMEs, consistent with previous research findings.

Furthermore, in this paper, we present initial evidence of a moderating effect based on gender in the ownership of SMEs. Women owners of SMEs moderate the relationship between environmental sustainability, open innovation, and innovation performance. The results indicate that women owners exhibit different managerial behaviors that can influence the impact of environmentally sustainable policies on the innovation strategy of SMEs.

In summary, this research highlights the positive influence of environmental sustainability on the open innovation practices of SMEs. The results emphasize the mediating role of innovation strategy and implementation in this relationship and underscore the importance of the moderating effect of women owners. The reported findings contribute to our understanding of the connections between environmental sustainability, open innovation, and innovation performance in SMEs.

## 7 Implications, limitations, and future lines of research

The results of this research have important implications both academically and practically. The findings demonstrate the significant impact of environmental sustainability on innovation practices, strategies, and implementation of SMEs. The high levels of interest among CEOs and reported adoption of such practices indicate recognition of their importance in the Ecuadorian business ecosystem, highlighting the need for CEOs to closely observe and adapt to the competitive environment by monitoring the actions of other companies.

Theoretically, the reported results contribute to our understanding the relationship between environmental sustainability and innovation in SMEs. In that sense, our findings contribute to the NRBV approach to understanding the sustainable business behavior of SMEs and the key effect of open innovation as a promoting mechanism to increase innovation performance in small business organizations. This fact should be considered by top management teams when implementing business strategies in SMEs. The findings reported herein emphasize the need for further research to explore the precise causes and effects of the identified moderating effect of CEO gender on the relationship between environmental strategy, open innovation, and innovation performance. Moreover, the presented investigation can be extended to larger corporations, where formalized organizational structures differ from those of SMEs.

For policymakers, the original findings presented herein are relevant to the promotion of public initiatives that can help managers of SMEs to increase green awareness that fosters pro-environmental practices with the aim of a long-term transition to protect the environment. Furthermore, we have demonstrated that environmental and governmental policies can help SMEs to increase open innovation, consequently improving the innovation performance of small and medium-sized companies.

However, the present research is subject to some limitations that open avenues for future studies. One limitation is the reliance on data obtained solely from CEOs, introducing the risk of biased responses. Although the Harman test was applied to mitigate this limitation, future research could benefit from considering the perspectives of departmental presidents and directors to capture diverse viewpoints within organizations. Additionally, the exploration of other cultural contexts and consideration of relational variables are crucial to gain a more comprehensive understanding of the subject. Lastly, given the cross-sectional nature of this study and because it was conducted at a specific moment in time, future longitudinal research would be valuable in assessing the robustness and stability of the results over time.

Overall, this research provides valuable insights into the impact of environmental sustainability on innovation practices in SMEs, highlighting the importance of considering different variables and expanding the scope of investigation in future studies.

## Author contribution statement

Conceived and designed the experiments: Paul Sarango-Lalangui.

Performed the experiments: Paul Sarango-Lalangui.

Analyzed and interpreted the data: Paul Sarango-Lalangui; Mauricio Castillo-Vergara; Omar Carrasco-Carvajal; Antonio Durendez.

Contributed reagents, materials, analysis tools or data: Paul Sarango-Lalangui; Mauricio Castillo-Vergara; Omar Carrasco-Carvajal; Antonio Durendez.

Wrote the paper: Paul Sarango-Lalangui; Mauricio Castillo-Vergara; Omar Carrasco-Carvajal; Antonio Durendez.

## Data availability statement

Data will be made available on request.

## Declaration of competing interest

The authors declare that they have no known competing financial interests or personal relationships that could have appeared to influence the work reported in this paper.
