# Supplementary material for: Impact of environmental sustainability on open innovation in SMEs: An empirical study considering the moderating effect of gender
Source: Heliyon. 2023 Sep 13;9(9):e20096. doi: 10.1016/j.heliyon.2023.e20096 (PMC10559863; doi:10.1016/j.heliyon.2023.e20096)
Supplement: Multimedia component 1 [file mmc1.docx]

| **RESEARCH PRESENTATION** |
| --- |
| This questionnaire has been designed to gather information on sustainability orientation in the context of Ecuadorian companies during 2019. The research is carried out in conjunction with researchers from the Universidad Técnica Particular de Loja, University of Barcelona and University of Extremadura, Spain.  Please answer all questions bearing in mind that there are no good or bad answers; try to indicate what reality is like in your company and not how you think it should be ideally.  We also wish to state that the information provided will be treated globally for research purposes, GUARANTEEING YOUR TOTAL CONFIDENTIALITY.  We thank you for your collaboration in this project, without which it would not be possible to achieve the objectives that we intend to achieve with it. |

| **BLOQUE I: Datos generales Nº Cuestionario:** | | | |
| --- | --- | --- | --- |
| **Enterprise name**  ____________________  **Industry**   1. 1. Technology 2. 2. Manufacturing 3. 3. Energy 4. 4. Water 5. 5. Construction 6. 6. Trade 7. 7. Transport 8. 8. Tourism 9. 9. Information   10. Financial | **Age of the company**   1. Less than 5 years 2. Between 5-10 years 3. Between 11-20 years 4. More than 20 years   **Position**   1. Director-General 2. Chief Financial Officer 3. Director of Human Resources 4. Administrative Director 5. Manager 6. Administrator   **Gender**   1. Male 2. Female | **Education**   1. Primary education 2. Secondary education 3. Technical studies 4. University studies 5. Diplomate 6. Master 7. DEA 8. PhD 9. Post-Doctor   **Age range**   1. Under 25 years of age 2. From 25 to 40 years old 3. From 41 years old to 65 years old 4. More than 65 years old | **Average number of employees:**   \| **Variable** \| **2017** \| **2018** \| \| --- \| --- \| --- \| \| Number of employees \|  \|  \| \| Number of women \|  \|  \| \| Number of men \|  \|  \|   **Country**  ____________________  **City**  ____________________ |

| **BLOQUE II: Competences sustainable entrepreneurship** |
| --- |
| The following is a series of statements related to competencies for sustainable entrepreneurship in the context of Ecuadorian enterprises. Please indicate your degree of agreement with each statement taking into account: 1 Totally disagree, 2 Disagree, 3 Neither agree nor disagree, 4 Agree and 5 Totally agree |
| \| **2. 1 Strategic management competence** \| VALUATION \| \| \| \| \| \| --- \| --- \| --- \| --- \| --- \| --- \| \| When it comes to achieving particular goals in relation to sustainability, I know whom to involve. \| 1 \| 2 \| 3 \| 4 \| 5 \| \| I am able to use a strategic way of working in sustainability related projects (designing, testing, implementing, evaluating). \| 1 \| 2 \| 3 \| 4 \| 5 \| \| I am able to monitor the sustainability performance of a company. \| 1 \| 2 \| 3 \| 4 \| 5 \| \| If I want to reach goals in relation to sustainability, I know which steps should be taken to be successful. \| 1 \| 2 \| 3 \| 4 \| 5 \| \| **2.2 Action competence** \| VALUATION \| \| \| \| \| \| I am very good at identifying opportunities for sustainable development. \| 1 \| 2 \| 3 \| 4 \| 5 \| \| I am able to motivate higher management in a company to invest in sustainability. \| 1 \| 2 \| 3 \| 4 \| 5 \| \| I know how social, environmental or societal challenges can be turned into opportunities for an organization/company. \| 1 \| 2 \| 3 \| 4 \| 5 \| \| I challenge non-sustainable ways of working in a company. \| 1 \| 2 \| 3 \| 4 \| 5 \| \| **2.3 Embracing diversity and Interdisciplinary** \| VALUATION \| \| \| \| \| \| I am able to actively involve stakeholders and experts from other disciplines in addressing sustainability issues. \| 1 \| 2 \| 3 \| 4 \| 5 \| \| I use the experiences, activities and values of various relevant stakeholders in addressing sustainability issues. \| 1 \| 2 \| 3 \| 4 \| 5 \| \| I am able to explain the importance of involving local stakeholders (e.g. in recruitment) for a company. \| 1 \| 2 \| 3 \| 4 \| 5 \| \| I am able to bring together economic, social and environmental conflicts of interest. \| 1 \| 2 \| 3 \| 4 \| 5 \| \| **2.4 Systems thinking competence** \| VALUATION \| \| \| \| \| \| I am able to identify the key operations of a company that have a negative impact on the environment or society. \| 1 \| 2 \| 3 \| 4 \| 5 \| \| I am able to identify key aspects of production chains and agricultural eco-systems. \| 1 \| 2 \| 3 \| 4 \| 5 \| \| I am able to analyze strengths and weaknesses of production chains and propose improvements to reduce the negative effects on the environment or society. \| 1 \| 2 \| 3 \| 4 \| 5 \| \| **2.5 Foresighted thinking** \| VALUATION \| \| \| \| \| \| I am able to construct and consider different directions for sustainability in the future \| 1 \| 2 \| 3 \| 4 \| 5 \| \| In analyzing and evaluating scenarios for action, I take both the impact on the local and the global scale into consideration. \| 1 \| 2 \| 3 \| 4 \| 5 \| \| I am able to identify risks and opportunities inherent in present and future developments. \| 1 \| 2 \| 3 \| 4 \| 5 \| \| In analyzing and evaluating scenarios for action, I take the impact on the short as well as the long term into consideration. \| 1 \| 2 \| 3 \| 4 \| 5 \| \| **2.6 Normative competence** \| VALUATION \| \| \| \| \| \| I am able to apply norms, values, targets and principles of sustainability to my own practice. \| 1 \| 2 \| 3 \| 4 \| 5 \| \| I know what is seen as ‘good sustainable practice’ in my field of study. \| 1 \| 2 \| 3 \| 4 \| 5 \| \| I know how to explain the decisions a company has made concerning sustainability. \| 1 \| 2 \| 3 \| 4 \| 5 \| \| I am willing to take initiative to make improvements in my own practice based on norms, values, targets and principles of sustainability. \| 1 \| 2 \| 3 \| 4 \| 5 \| \| **2.7 Interpersonal competence** \| VALUATION \| \| \| \| \| \| In a personal conflict, I am able to take the others’ perspective and really understand his or her point of view. \| 1 \| 2 \| 3 \| 4 \| 5 \| \| I am patient and sensitive to someone who “lets off steam” in complex issues. \| 1 \| 2 \| 3 \| 4 \| 5 \| \| I am able to feel to what extent stakeholders are willing to cooperate in a project. \| 1 \| 2 \| 3 \| 4 \| 5 \| |
|  |
| **BLOQUE III: Environmental orientation** |
| The following is a series of statements related to environmental orientation in the context of Ecuadorian companies. Please indicate your degree of agreement with each statement by taking into account: 1 Totally disagree, 2 Disagree, 3 Neither agree nor disagree, 4 Agree and 5 Totally agree.   \| **3.1. Internal Environmental Orientation** \| VALUATION \| \| \| \| \| \| --- \| --- \| --- \| --- \| --- \| --- \| \| Our firm makes concerted efforts to let every employee understand the importance of environmental preservation. \| 1 \| 2 \| 3 \| 4 \| 5 \| \| Our firm has clear policy statements urging environmental awareness in every area of operations. \| 1 \| 2 \| 3 \| 4 \| 5 \| \| Environmental preservation is highly valued by our firm members. \| 1 \| 2 \| 3 \| 4 \| 5 \| \| Environmental preservation is a central corporate value of our firm. \| 1 \| 2 \| 3 \| 4 \| 5 \| \| Environmental issues are not very relevant to the major function of our firm. \| 1 \| 2 \| 3 \| 4 \| 5 \| \| We try to promote environmental preservation as major goal across all departments. \| 1 \| 2 \| 3 \| 4 \| 5 \| \| **3.2. External Environmental Orientation** \| VALUATION \| \| \| \| \| \| In our firm, environmental preservation is largely an issue of maintaining a good public image. \| 1 \| 2 \| 3 \| 4 \| 5 \| \| The developments in the natural environment affect our firm's business activities. \| 1 \| 2 \| 3 \| 4 \| 5 \| \| The financial well-being of our firm depends on the state of the natural environment. \| 1 \| 2 \| 3 \| 4 \| 5 \| \| Environmental preservation is vital to our firm's survival. \| 1 \| 2 \| 3 \| 4 \| 5 \| \| Various external stakeholders expect our firm to preserve the environment. \| 1 \| 2 \| 3 \| 4 \| 5 \| \| Our firm strives for an image of environmental responsibility. \| 1 \| 2 \| 3 \| 4 \| 5 \| \| Our firm’s responsibility to its customers, stockholders, and employees is more important than our responsibility toward environmental preservation. \| 1 \| 2 \| 3 \| 4 \| 5 \| |

| **BLOQUE IV: Orientation social entrepreneurship** |
| --- |
| The following is a series of statements related to the Orientation to social entrepreneurship in the context of Ecuadorian companies. Please indicate your degree of agreement with each statement taking into account: 1 Totally disagree, 2 Disagree, 3 Neither agree nor disagree, 4 Agree and 5 Totally agree. |
| \| **4.1. Social innovativeness** \| VALUATION \| \| \| \| \| \| --- \| --- \| --- \| --- \| --- \| --- \| \| Social innovation is important for our company \| 1 \| 2 \| 3 \| 4 \| 5 \| \| We invest heavily in developing new ways to increase our social impact or to serve our beneficiaries \| 1 \| 2 \| 3 \| 4 \| 5 \| \| In our company, new ideas to solve social problems come up very frequently \| 1 \| 2 \| 3 \| 4 \| 5 \| \| **4.2. Social risk-taking** \| VALUATION \| \| \| \| \| \| We are not afraid to take substantial risks when serving our social purpose \| 1 \| 2 \| 3 \| 4 \| 5 \| \| Bold action is necessary to achieve our company’s social mission \| 1 \| 2 \| 3 \| 4 \| 5 \| \| We avoid the cautious line of action if social opportunities might be lost that way \| 1 \| 2 \| 3 \| 4 \| 5 \| \| **4.3. Socialness** \| VALUATION \| \| \| \| \| \| The objective to accomplish our social mission precedes the objective to generate a profit \| 1 \| 2 \| 3 \| 4 \| 5 \| \| Our organization places a strong focus on partnerships with other organizations and/or governments in order to ensure a greater and accelerated accomplishment of the social mission. \| 1 \| 2 \| 3 \| 4 \| 5 \| \| We set ourselves ambitious goals in regard to sustainability and incorporate them in all strategic decisions. \| 1 \| 2 \| 3 \| 4 \| 5 \| \| **4.4. Social proactiveness** \| VALUATION \| \| \| \| \| \| We aim at being at the forefront of making the world a better place \| 1 \| 2 \| 3 \| 4 \| 5 \| \| Our organization has a strong tendency to be ahead of others in addressing its social mission \| 1 \| 2 \| 3 \| 4 \| 5 \| \| We typically initiate actions which other social enterprises/social entrepreneurs copy \| 1 \| 2 \| 3 \| 4 \| 5 \| |
|  |

| **BLOQUE V: Performance** |
| --- |
| The following is a series of statements related to performance in the context of Ecuadorian companies. Please indicate your degree of agreement with each statement by taking into account: 1 Totally disagree, 2 Disagree, 3 Neither agree nor disagree, 4 Agree and 5 Totally agree. |
| \| **5.1 . Financial performance** \| VALUATION \| \| \| \| \| \| --- \| --- \| --- \| --- \| --- \| --- \| \| Our investors are satisfied with return on investment. \| 1 \| 2 \| 3 \| 4 \| 5 \| \| Our company has Earnings growth \| 1 \| 2 \| 3 \| 4 \| 5 \| \| Our company has Sales growth \| 1 \| 2 \| 3 \| 4 \| 5 \| \| Our company has improved market share change \| 1 \| 2 \| 3 \| 4 \| 5 \| \| **5.2. Social performance** \| VALUATION \| \| \| \| \| \| Our beneficiaries are satisfied with our services. \| 1 \| 2 \| 3 \| 4 \| 5 \| \| We help mobilize interest for additional social welfare initiatives. \| 1 \| 2 \| 3 \| 4 \| 5 \| \| The output provided by our organization has a significant impact on general well-being. \| 1 \| 2 \| 3 \| 4 \| 5 \| \| Our organization is on a good path to accomplish its social mission. \|  \|  \|  \|  \|  \| \| **5.3 Green Innovation Performance** \| VALUATION \| \| \| \| \| \| Our enterprise has bound itself strongly to developing new things, to product development and to innovations. \| 1 \| 2 \| 3 \| 4 \| 5 \| \| Our enterprise has introduced many new products or services on the market. \| 1 \| 2 \| 3 \| 4 \| 5 \| \| The changes in products or services we offer are typically significant. \| 1 \| 2 \| 3 \| 4 \| 5 \| \| Eco-balancing in the whole enterprise \| 1 \| 2 \| 3 \| 4 \| 5 \| \| Adoption of annual and biannual sustainability reports \| 1 \| 2 \| 3 \| 4 \| 5 \| \| Institutionalization of a board of sustainability \| 1 \| 2 \| 3 \| 4 \| 5 \| \| Implementation of sustainability division \| 1 \| 2 \| 3 \| 4 \| 5 \| \| Establishing of sustainability cooperation \| 1 \| 2 \| 3 \| 4 \| 5 \| \| Institutionalization of intranet/platforms/network for the exchange of sustainability related information \| 1 \| 2 \| 3 \| 4 \| 5 \| \| Stakeholder integration in environmental themes \| 1 \| 2 \| 3 \| 4 \| 5 \| \| The company chooses the materials of the product that produce the least amount of pollution for conducting the product development or design. \| 1 \| 2 \| 3 \| 4 \| 5 \| \| The company chooses the materials of their products that consume the least amount of energy and resources for conducting the product development or design. \| 1 \| 2 \| 3 \| 4 \| 5 \| \| The company uses the fewest amount of materials to comprise their products for conducting the product development or design. \| 1 \| 2 \| 3 \| 4 \| 5 \| \| The company would circumspectly evaluate whether their products are easy to recycle, reuse, and decompose for conducting the product development or design. \| 1 \| 2 \| 3 \| 4 \| 5 \| \| The manufacturing process of the company effectively reduces the emission of hazardous substances or wastes. \| 1 \| 2 \| 3 \| 4 \| 5 \| \| The manufacturing process of the company effectively recycles wastes and emission that can be treated and re-used. \| 1 \| 2 \| 3 \| 4 \| 5 \| \| The manufacturing process of the company effectively reduces the consumption of water, electricity, coal, or oil. \| 1 \| 2 \| 3 \| 4 \| 5 \| \| The manufacturing process of the company effectively reduces the use of raw materials. \| 1 \| 2 \| 3 \| 4 \| 5 \| |
|  |

| **BLOQUE VI: Sustainable entrepreneurship team.** |
| --- |
| Below are a number of statements related to the Sustainable Entrepreneurship Team in the context of Ecuadorian businesses. Please indicate your degree of agreement with each statement taking into account: 1 Totally disagree, 2 Disagree, 3 Neither agree nor disagree, 4 Agree and 5 Totally agree. |
| \| **6.1. Intrinsic Motivation** \| VALUATION \| \| \| \| \| \| --- \| --- \| --- \| --- \| --- \| --- \| \| We are motivated by help others and the surrounding society, as well as care for the environment \| 1 \| 2 \| 3 \| 4 \| 5 \| \| We are willing to engage in challenging work \| 1 \| 2 \| 3 \| 4 \| 5 \| \| We are passionate about solving problems creatively \| 1 \| 2 \| 3 \| 4 \| 5 \| \| **6.2. Extrinsic Motivation** \| VALUATION \| \| \| \| \| \| We hope to make a good profit by \| 1 \| 2 \| 3 \| 4 \| 5 \| \| We hope to solve social or environment problems by entrepreneurship \| 1 \| 2 \| 3 \| 4 \| 5 \| \| We hope that our work achievement can be recognized by the public \| 1 \| 2 \| 3 \| 4 \| 5 \| \| **6.3. Opportunity Recognition** \| VALUATION \| \| \| \| \| \| We can identify products or services which people want in the future \| 1 \| 2 \| 3 \| 4 \| 5 \| \| We can perceive unmet consumer needs from social or environment problem \| 1 \| 2 \| 3 \| 4 \| 5 \| \| We can look for products or services that provide real social or environmental benefit \| 1 \| 2 \| 3 \| 4 \| 5 \| \| **6.4. Knowledge Resources Acquirement** \| VALUATION \| \| \| \| \| \| We can acquire knowledge and information for Research & Development of new products or services \| 1 \| 2 \| 3 \| 4 \| 5 \| \| We can acquire knowledge and information for new market development \| 1 \| 2 \| 3 \| 4 \| 5 \| \| We can acquire knowledge and information for production and operation \| 1 \| 2 \| 3 \| 4 \| 5 \| \| **6.5. Sustainable Entrepreneurship Outcome** \| VALUATION \| \| \| \| \| \| Our outcome has social, economic, and environmental value \| 1 \| 2 \| 3 \| 4 \| 5 \| \| Our outcome can gain major market share in the future \| 1 \| 2 \| 3 \| 4 \| 5 \| \| Our outcome can promote social change to some extent \| 1 \| 2 \| 3 \| 4 \| 5 \| |
|  |

| **BLOQUE VII: Corporate environmentalism** |
| --- |
| Below are a series of statements related to corporate environmentalism in the context of Ecuadorian companies. Please indicate your degree of agreement with each statement taking into account: 1 Totally disagree, 2 Disagree, 3 Neither agree nor disagree, 4 Agree and 5 Totally agree. |
| \| **7.1. Public concern** \| VALUATION \| \| \| \| \| \| --- \| --- \| --- \| --- \| --- \| --- \| \| ﻿Our customers feel that environmental protection is a critically important issue facing the world today. \| 1 \| 2 \| 3 \| 4 \| 5 \| \| Our customers are increasingly demanding environmentally friendly products and services. \| 1 \| 2 \| 3 \| 4 \| 5 \| \| The public is more worried about the economy than about environmental protection. \| 1 \| 2 \| 3 \| 4 \| 5 \| \| Our customers expect our firm to be environmentally friendly. \| 1 \| 2 \| 3 \| 4 \| 5 \| \| **7.2. Regulatory forces** \| VALUATION \| \| \| \| \| \| Regulation by government agencies has greatly influenced our firm’s environmental strategy. \| 1 \| 2 \| 3 \| 4 \| 5 \| \| Environmental legislation can affect the continued growth of our firm. \| 1 \| 2 \| 3 \| 4 \| 5 \| \| Stricter environmental regulation is a major reason why our firm is concerned about its impact on the natural environment. \| 1 \| 2 \| 3 \| 4 \| 5 \| \| Tougher environmental legislation is required so that only firms that are environmentally responsible will survive and grow. \| 1 \| 2 \| 3 \| 4 \| 5 \| \| Our firm’s environmental efforts can help shape future environmental legislation in our industry. \| 1 \| 2 \| 3 \| 4 \| 5 \| \| Our industry is faced with strict environmental regulation. \| 1 \| 2 \| 3 \| 4 \| 5 \| \| **7.3. Competitive advantage** \| VALUATION \| \| \| \| \| \| Being environmentally conscious can lead to substantial cost advantages for our firm. \| 1 \| 2 \| 3 \| 4 \| 5 \| \| Our firm has realized significant cost savings by experimenting with ways to improve the environmental quality of our products and processes. \| 1 \| 2 \| 3 \| 4 \| 5 \| \| By regularly investing in research and development on cleaner products and processes, our firm can be a leader in the market. \| 1 \| 2 \| 3 \| 4 \| 5 \| \| Our firm can enter lucrative new markets by adopting environmental strategies. \| 1 \| 2 \| 3 \| 4 \| 5 \| \| Our firm can increase market share by making our current products more environmentally friendly. \| 1 \| 2 \| 3 \| 4 \| 5 \| \| Reducing the environmental impact of our firm’s activities will lead to a quality improvement in our products and processes. \| 1 \| 2 \| 3 \| 4 \| 5 \| \| At our firm we make every effort to link environmental objectives with our other corporate goals. \| 1 \| 2 \| 3 \| 4 \| 5 \| \| Our firm is engaged in developing products and processes that minimize environmental impact. \| 1 \| 2 \| 3 \| 4 \| 5 \| \| Environmental protection is the driving force behind our firm’s strategies. \| 1 \| 2 \| 3 \| 4 \| 5 \| \| Environmental issues are always considered when we develop new products. \| 1 \| 2 \| 3 \| 4 \| 5 \| \| Our firm develops products and processes that minimize environmental impact. \| 1 \| 2 \| 3 \| 4 \| 5 \| |
|  |

**Stamp of the enterprise E-mail of the enterprise** (if you want to receive the sectorial study).

(only for the purpose of verifying the work

of the information collectors) **IMPORTANT: PUT UPPERCASE LETTERS**

…………………………………………………@…………………………………………………
