# Supplementary material for: Impact of environmental sustainability on open innovation in SMEs: An empirical study considering the moderating effect of gender
Source: Heliyon. 2023 Sep 13;9(9):e20096. doi: 10.1016/j.heliyon.2023.e20096 (PMC10559863; doi:10.1016/j.heliyon.2023.e20096)
Supplement: Multimedia component 2 [file mmc2.docx]

ESTUDIO SOBRE SUSTENTABILIDAD EN PYMES DEL ECUADOR

Consentimiento Informado

Nos gustaría invitarle a participar en el estudio “SUSTENTABILIDAD EN PYMES DEL ECUADOR". Este estudio tiene como objetivo determinar empíricamente el impacto de la sustentabilidad en las pequeñas y medianas empresas del Ecuador, y los impactos que estas prácticas tienen en el desempeño de las empresas.

Si usted acepta colaborar en el estudio se le pedirá que complete un cuestionario de preguntas que le tomarán aproximadamente 20 minutos como máximo. La participación en esta encuesta es totalmente voluntaria. Usted tiene el derecho a abandonarla sin necesidad de dar ningún tipo de explicación y sin que ello signifique ningún perjuicio para usted ni para la empresa que representa, como tampoco la pérdida de ningún derecho.

Toda la información que aporte será tratada de manera confidencial, de acuerdo con la ley orgánica de protección de datos personales. Por las características de la investigación, aseguramos que usted no corre peligro alguno, ya que, los datos recogidos (encuestas y consentimientos) serán resguardados en la base de datos como archivo Excel.

En caso de que desee realizar cualquier tipo de consulta, puede comunicarse con el investigador responsable Paul Sarango al correo posarango@utpl.edu.ec.

Para poder continuar la encuesta online, por favor indíquenos si:

¿Acepta participar en este estudio, confirmando que ha sido informado sobre su propósito y condiciones?

Si

No
